# Patients' Perception Regarding the Communicative Capabilities of Portuguese Family Doctors: A Cross-Sectional Study

**DOI:** 10.7759/cureus.100336

**Published:** 2025-12-29

**Authors:** Catarina A Gonçalves, Catarina F Alves, Catarina S Alves, Rita Coutinho, Ludovina Rocha, Bárbara Moreira, Pedro Seabra

**Affiliations:** 1 USF (Unidade de Saúde Familiar) Pedras Rubras, ULS (Unidade Local de Saúde) São João, Maia, PRT; 2 USF (Unidade de Saúde Familiar) Vil'Alva, ULS (Unidade Local de Saúde) Médio Ave, Santo Tirso, PRT; 3 USF (Unidade de Saúde Familiar) Alpendorada|Tabuado, ULS (Unidade Local de Saúde) Tâmega e Sousa, Marco de Canaveses, PRT; 4 USF (Unidade de Saúde Familiar) São Neutel, ULS (Unidade Local de Saúde) Trás-os-Montes e Alto Douro, Chaves, PRT; 5 USF (Unidade de Saúde Familiar) Caminhos do Cértoma, ULS (Unidade Local de Saúde) Coimbra, Coimbra, PRT

**Keywords:** communication, doctor-patient relation, family practice, interpersonal relations, primary care

## Abstract

Introduction

Effective communication is the cornerstone of the doctor-patient relationship, playing a fundamental role in fostering a collaborative partnership between patients and their family physicians. A variety of factors, including gender and socioeconomic status, have been identified as influencing the quality of communication within this dyadic interaction. Our study aimed to assess how patients perceive their doctors' communicative behaviors across different regions of the country, considering multiple dimensions of doctor-patient communication, as well as the patients’ personal and sociodemographic characteristics.

Materials and methods

This cross-sectional observational study involved the administration of the Doctor Communication Behavior Questionnaire (DCBQ-QCCM) to a representative sample of 439 patients, followed by statistical analysis using the Mann-Whitney test and the Kruskal-Wallis test to detect potential differences in perceptions based on various sociodemographic and regional variables.

Results

The DCBQ-QCCM demonstrated excellent internal consistency, with a Cronbach’s alpha coefficient of 0.964. The majority of participants agreed with the questionnaire statements. Items with the lowest levels of agreement included “Does your doctor ask for your opinion during consultations?” and “Does your doctor ask you to explain the proposed treatment in your own words?”. The domain with the highest level of agreement was “comprehension” (89.8%), followed by “verbal communication” (81.8%), “control” (80%), and “encouragement” (78.6%). Statistically significant differences were found in the perceptions of communication based on regional differences (rural versus urban - p = 0.015), type of physician (family doctor versus other physician - p = 0.027), and gender within the doctor-patient dyad (p = 0.004).

Discussion

Our findings suggest that communication is perceived to be better in rural areas compared to urban settings. Moreover, female physicians were perceived to demonstrate superior communication skills compared to their male counterparts. Notably, the greatest communication challenges appear to arise in interactions between female patients and male doctors.

Conclusion

Overall, patients expressed a positive perception of the communication skills of their family doctors. However, it is essential to adopt an active approach that emphasizes “giving a voice to the patient” to further enhance communication in the clinical setting.

## Introduction

Communication is a valuable tool in the clinical practice of the specialty of Family and Community Medicine. This medical specialty emphasizes a biopsychosocial approach to the patient, which is fundamental for understanding the biological, psychological, and social dimensions of the patient. Thus, communication acquires special importance in the different phases of the doctor-patient relationship, relationship with the patient's relatives, collection of clinical information, transmission of information on medical therapies, disease prevention, and health education [1].

Physician-patient communication is based on verbal and nonverbal interaction, always interpreted by the patient. It is believed that only 7% of communication is transmitted verbally and 38% is transmitted nonverbally through expressions and gestures [2]. 

Nonverbal communication is the most influential in the first contact between these two interlocutors, but both play a crucial role in the establishment of a relationship of trust between physician and patient [1]. It is important to note that the physician's individual communication style and his own sociocultural context have a great influence on this relationship, and it is essential that he acquires communication skills to positively manage the patient's concerns, wishes, and doubts [1]. Through communication, the physician becomes aware of the individual characteristics of each patient, his experiences, and the social and cultural context in which he is immersed [1]. In this way, it is easier to create an empathic relationship with the patient and to find appropriate strategies to increase the effectiveness of interventions in health education, disease prevention, control of pre-existing diseases, and diagnosis of new pathologies [1]. Experimental studies indicate that the medical history obtained through effective communication provides about 85% of the information needed to make a diagnosis, while the physical examination and complementary means of diagnosis provide only about 10% and 5%, respectively [1].

The effective communicative process between physician and patient positively influences patient satisfaction with the consultation and adherence to medical treatment, which is even more relevant in chronic diseases that require prolonged patient involvement [3]. Satisfaction is related to the physician's ability to respond to the patient's concerns and to the fact that there is a patient-centered medical practice, where there is a sharing of power and responsibility for the patient's health between physician and patient, forming a therapeutic alliance [1,2]. It is known that patients who are more satisfied and have greater trust in their physician tend to adhere more to the plan proposed by the physician and attend fewer medical appointments [1].

It has been proven, after the analysis of several studies, that demographic and socioeconomic factors influence physician-patient communication. Patients from lower social classes tend to receive less information and emotional support and have less involvement in decisions, whereas patients from higher social classes, who communicate more actively, receive more information and are more involved in the relationship with the physician. In addition, the perception of physicians' communication competence may vary according to the gender and educational level of patients [3-5].

Although several factors have been documented as influencing the quality of communication in the doctor-patient dyad, there is a paucity of evidence regarding these factors in the context of the Portuguese population. This study aimed to 1) evaluate patients’ perception of physician communication skills using the Doctor Communication Behavior Questionnaire (DCBQ-QCCM) and 2) determine if these perceptions are associated with factors such as geographic location (rural/urban), doctor-patient gender dyads, and type of physician (family doctor versus other). Overall perceptions of physicians’ communication skills were expected to be positive, though comparatively lower among patients with lower educational attainment and those living in rural areas. 

This article was previously presented as an oral communication at the 9th EYFDM Forum 2025 on April 26, 2025.

## Materials and methods

An observational and cross-sectional study was conducted, covering 439 patients evaluated in General and Family Medicine consultations in five Family Health Units (FHUs) in northern and central Portugal.

The study included patients aged 18 years or older, enrolled in the FHUs (N = 41,616, data referring to December 2023), who attended scheduled appointments (not urgent). A significant sample of this population was calculated using the Raosoft® program (Raosoft, Inc., Seattle, WA) (considering a prevalence of 50%, a precision level of 5%, and a confidence interval of 95%), totaling 381 patients. The authors excluded illiterate patients without an accompanying person able to help complete the questionnaires, patients with cognitively incapacitating diseases, and poorly completed questionnaires. The first two exclusion criteria were ensured by personal delivery of the questionnaires by the administrative staff. 

Immediately after their scheduled consultations, over a four-week period in 2024, the first 10 patients who met the criteria to attend each FHU per day (totaling 50 patients per day across all FHUs) were provided with self-completion questionnaires distributed by the administrative staff, constituting a convenience sample. The completed questionnaires were then anonymously placed in a locked box provided for that purpose. Physicians were intentionally kept unaware of the distribution timeframe to minimize potential bias. 

The questionnaire included three parts: part 1 regarding sociodemographic data; part 2 regarding consultation data; and part 3 was the DCBQ-QCCM [3]. Parts 1 and 2 of the questionnaire were elaborated by the authors. 

The variables studied included patients’ age, gender, educational level, and area of residence, as well as the gender of the physician who performed the scheduled consultation and the physician’s type (family doctor versus other).

The DCBQ-QCCM was originally developed and validated in Brazilian Portuguese in 2010 by Luciana Croitor [3]. This validation study demonstrated good psychometric properties, including satisfactory internal consistency (Cronbach’s alpha = 0.87 for the total scale and acceptable alpha coefficients for most domains), a five-factor structure explaining approximately 55% of the total variance, and evidence of external validity through its association with the Patient-Practitioner Orientation Scale (PPOS) [3]. The DCBQ-QCCM consists of 23 questions, divided into five domains: challenge (three questions), encouragement and praise (three questions), nonverbal support (five questions), understanding and friendly relationship (10 questions), and control (two questions). Each question presents five response options, numbered from 1 to 5, where 1 means “Never” and 5 means “Always”, indicating the frequency with which each action occurs in practice. 

In the present study, the original 23-item version of the DCBQ-QCCM was used without any adaptation of items or response format, and its use was authorized by Croitor. Scores were calculated using mean values. For each participant, the total score was obtained by calculating the arithmetic mean of the responses to all 23 items, resulting in a total score ranging from 1 to 5. Domain scores were calculated the same way. A mean score equal to or greater than 4 (“Frequently”) was used by the authors as the threshold to indicate good overall communication, and the same cutoff was applied to domain-specific scores to indicate strength in each communicative domain.

Statistical analysis was performed using SPSS® Statistics software, version 29 (IBM Corp., Armonk, NY). Categorical variables were evaluated using absolute and relative frequencies, and continuous variables using mean, standard deviation, median, and interquartile range when applicable.

Internal consistency of the DCBQ-QCCM in this sample was assessed using Cronbach’s alpha for the total score. This coefficient was calculated on the main study dataset (N = 439) to verify reliability in the present context.

The normality of the global communication score (derived from the DCBQ-QCCM) was formally assessed using the Shapiro-Wilk test, which demonstrated a non-normal distribution (W = 0.859; p < 0.001). Given the ordinal nature of the Likert-based scale and its non-normal distribution, non-parametric tests were selected. The Mann-Whitney test was used to compare the global communication score between dichotomous variables, namely patient gender, physician gender, rural versus urban residence, and physician type (family doctor versus other). Because the physician-patient gender dyad comprises more than two distinct categories as well as years as the family physician and level of education, the Kruskal-Wallis test was applied to compare differences across these groups, as the Mann-Whitney test is only appropriate for two-group comparisons. A p-value equal to or less than 0.05 (p ≤ 0.05) was considered statistically significant. 

Ethical considerations

The study was approved by the Board of Directors and received approval from the Health Ethics Committee of the São João Local Health Unit, with the specific reference CE-103-2024.

## Results

Over the four-week study period, a total of 1000 questionnaires were distributed, of which 500 were returned, yielding a response rate of 50.0%. Of these, 439 questionnaires (87.8%) were included in the analysis, while the remaining ones were excluded due to incomplete completion.

Tables 1, 2 present the sociodemographic data of the sample, corresponding to the first part of the questionnaire applied, and the data on the characteristics of the consultation, respectively.

**Table 1:** Sociodemographic Data of the Sample (Total N = 439) N: number of people; %: percentage.

Sociodemographic Data		N	%
Age group (years)	<30	60	13.7
	30-39	84	19.1
	40-49	83	18.9
	50-59	94	21.4
	60-69	69	15.7
	≥70	49	11.2
Patient gender	Female	300	68.3
	Male	139	31.7
Level of education	Illiterate	1	0.2
	Elementary school	99	22.5
	Middle school	115	26.2
	High school	135	30.8
	Higher education	89	20.3
Place of residence	Rural	168	38.3
	Urban	271	61.7

**Table 2:** Characteristics of the Consultation N: number of people; %: percentage. ^1^Total N = 439. ^2^Total N = 366.

Characteristics of the Consultation		N	%
Doctors' gender^1^	Female	285	64.9
	Male	154	35.1
Who was the doctor^1^	Family doctor	366	83.4
	Other	73	16.7
First contact with the consulting doctor^1^	Yes	82	18.7
	No	357	81.3
Years of follow-up with the family doctor^2^	<1 year	58	15.8
	1-3 years	48	13.1
	3-5 years	62	16.9
	>5 years	198	54.1
Doctor-patient dyad^1^	Female-female	199	45.3
	Male-male	53	12.1
	Female-male	101	23.0
	Male-female	86	19.6

Of the analyzed questionnaires, the mean age of patients was 48.6 years (SD = 16.7), with a median age of 49 years (IQR = 26; Q1 = 35, Q3 = 61). A higher proportion of responses was obtained from female patients (N = 285, 68.3%). Most patients had completed high school or higher education (N = 224, 51.1%) and resided in urban areas (according to the Rural Development Program 2020 classification) [6].

In the sample analyzed, most patients consulted with female physicians (N = 285, 64.9%), and the most frequent physician-patient dyad was female-female (N = 199, 45.3%). Of the patients who consulted their family physician (N = 366, 83.4%), for 42 (11.5%), it was the first consultation with this professional, and only 153 patients (34.9%) mentioned a regular consultation every six months due to their chronic diseases.

Regarding the third part of the questionnaire applied, which corresponds to the Likert scales, the internal consistency of the DCBQ-QCCM total score in this sample was excellent (Cronbach’s alpha = 0.964), confirming the reliability of the instrument in this primary care context. This Cronbach’s alpha was calculated on the main study sample and complements the psychometric properties previously reported in the original validation study.

The average mean score of the total scale was 4.36 (SD = 0.66; median = 4.52), corresponding to an overall good perception of communication according to the threshold of ≥4.0. The majority of the sample population reported that their doctors always performed the communicative behaviors listed in the questionnaire (mode of all items: 5), and in the overall analysis of the scale, most participants (N = 226, 51.5%) indicated that their doctor always does the behaviors stated; only 34 (7.7%) reported that these behaviors were rarely or never performed. The behaviors reported as least frequently observed were “Does your doctor ask for your opinion during consultations?” (mean: 3.86) and “Does he/she ask you to explain in your own words the proposed treatment?” (mean: 3.99); all the other statements obtained means above 4.1, with the highest frequencies reported for “Does he/she explain again what you did not understand?” and “Does he/she listen to you if you have something to say?” (mean of 4.59 and 4.58, respectively).

When analyzing the different domains of the questionnaire, the comprehension component showed better results (89.8% were classified as 4 or 5), followed by the nonverbal component (81.8%), control (80.0%), and, finally, stimulus (78.6%).

Statistically significant variation in global scores was observed in terms of the physician's communication skills perceived by patients across multiple variables. 

To better illustrate the direction and magnitude of the observed differences, Table 3 summarizes the mean and median global communication scores across subgroups. All analyses were conducted on the full sample (N = 439), except for years of follow-up with the family physician, which was analyzed only among consultations performed by family physicians (N = 366).

**Table 3:** Mean and Median Global Communication Scores for the Analyzed Variables

Variable	Group	Mean	Median
Region	Rural	4.46	4.65
	Urban	4.30	4.43
Doctor type	Family doctor	4.41	4.57
	Other physician	4.28	4.48
Consultation frequency	Semi-annual	4.45	4.65
	Not semi-annual	4.32	4.48
Gender dyad	Female patient-female doctor	4.45	4.61
	Male patient-male doctor	4.44	4.57
	Female patient-male doctor	4.09	4.26
	Male patient-female doctor	4.43	4.57
Educational level	Illiterate	4.52	4.52
	Elementary school	4.34	4.65
	Middle school	4.27	4.43
	High school	4.39	4.61
	Higher education	4.46	4.57
First contact with the consulting doctor	Yes	4.30	4.57
	No	4.38	4.52
Years of follow-up with the family doctor	<1 year	4.45	4.72
	1-3 years	4.37	4.59
	3-5 years	4.27	4.35
	>5 years	4.41	4.55

Using the Mann-Whitney test, differences were identified between rural and urban patients (p = 0.015), between those consulting their family doctor versus another physician (p = 0.027), and between patients attending semi-annual consultations and those who did not (p = 0.013). As the physician-patient gender dyad comprises four categories, differences were assessed using the Kruskal-Wallis test, which also indicated a significant effect (p = 0.004).

Communication was perceived as more positive among rural patients compared with urban patients, among those consulting their family doctor rather than another physician, and among patients attending semi-annual consultations. In terms of the physician-patient gender dyad, the lowest scores were observed in female patient-male doctor interactions.

No statistically significant variation in global communication scores was observed for the patient's educational level or years of follow-up with the family physician (Kruskal-Wallis p = 0.517 and p = 0.407, respectively), as well as, if it was the first contact with the family physician or not (Mann-Whitney p = 0.440).

## Discussion

In this study, the authors aimed to evaluate patients’ views of physician communication in Portuguese primary healthcare and investigate possible influencing factors. Considering our threshold of ≥4.0 to be indicative of a generally positive perception of family physicians’ communication skills, the high overall mean score observed in our sample supports the interpretation of a satisfactory level of doctor-patient interaction in primary care. Several factors were found to potentially influence patients’ perception of doctors' communication behaviors, such as the patient’s place of residence, the physician's gender, the type of physician, and the continuity of care, highlighting the complexity of interpersonal dynamics in primary healthcare consultations. 

Effective communication is a fundamental part of the doctor-patient relationship and plays a vital role in delivering high-quality healthcare. When communication is strong, patients are more satisfied, better understand their condition and treatment, and are more likely to follow medical advice [7,8]. In our study, most patients reported a generally positive perception of their family physician’s communication skills and behaviors, in contrast to existing research indicating that physicians often overestimate their own communicative abilities [7].

Contrary to the initial expectation that patients with lower educational attainment or those living in rural areas would perceive poorer communication, our results suggest that communication was actually perceived as more positive in rural settings. No relationship was found between the patients’ level of education and the perception of physicians’ communication skills. 

Regarding the genders of the physician-patient dyads, there seems to be a better perception of the fulfillment of good communication behaviors/skills, in general, by female physicians with female patients, followed by female-male and male-male dyads, with the greatest difficulties encountered between male physicians and female patients. The female patient-male physician dyad showed the lowest mean and median global communication scores (Table 3), reinforcing that this interaction represents the most challenging communication dynamic. Communication is a skill that can be taught and improved, and investing in such training is worthwhile [8]. These findings suggest that tailored communication training could be especially advantageous for male physicians, with a focus on improving interactions with female patients.

Family doctors seem to be better at taking the necessary steps to achieve better communication with their patients compared to other physicians (including residents), and patients with semiannual follow-up (N = 153, 34.9%) seem to have a better perception of the physician's communication skills compared to patients without such follow-up. 

Of the patients whose consultation was performed by the family physician (N = 366), 260 (71.0%) had at least three years of follow-up, which could justify the lack of a statistically significant relationship in this variable.

It is important to acknowledge that this study presents several limitations. First, participation/non-response bias may have influenced the results, as only half of the distributed questionnaires were returned. Patients who chose to participate may have held a more favorable perception of their physicians’ communication behaviors, while less satisfied patients may have been less inclined to respond. In addition, social desirability bias may have contributed to the high scores observed, particularly given the clinical setting in which data were collected. Furthermore, because data were self-reported, patients’ subjective opinions may not accurately reflect physicians’ actual communication behaviors.

Additionally, the use of a convenience sample may have introduced selection bias and limited the generalizability of the findings. Patients who regularly attend consultations and have ongoing relationships with their physicians may perceive communication more positively, and dissatisfied patients may have been less likely to participate. Consequently, although statistically significant associations were detected, the results may reflect the characteristics of the specific group of patients who agreed to participate rather than those of the wider Portuguese primary care population. Therefore, these findings should be interpreted with caution, and future studies employing probabilistic sampling methods are needed to confirm these patterns.

Moreover, the high frequency of maximum scores observed across the items may suggest the presence of a ceiling effect, which could have limited the sensitivity of the DCBQ-QCCM to detect more nuanced differences between groups. This effect is particularly relevant in high-performing clinical contexts, such as primary healthcare settings with established physician-patient relationships. Although statistically significant associations were still identified, these findings should be interpreted with caution, as the magnitude of differences may be underestimated.

Conversely, first-time patients were underrepresented (N = 42, 11.5%), which may underestimate communication challenges in unfamiliar clinical encounters. Furthermore, the cross-sectional design captures patients’ perceptions at a single point in time, limiting causal inferences about the factors influencing communication quality. 

Another important aspect is that the distribution of patients evaluated in the different categories is not uniform, nor is their distribution in the national territory, which could influence the results. The distribution of patients in rural and urban areas was based on the PDR 2020 classification. However, this division into rural and urban areas is not simple and has different definitions, which could contribute to an incorrect classification bias. This classification also includes 2014 data that may be outdated. 

Finally, the exclusion of patients who are illiterate or cognitively disabled may have skewed the sample toward healthier and more educated individuals, potentially underrepresenting more vulnerable populations and thus limiting the generalizability of the findings.

To address these limitations and improve the generalizability of the results, future studies should include primary healthcare centers from a wider range of regions across the country and employ random sampling over a longer data collection period, rather than relying on convenience sampling. Future research may benefit from the use of instruments with greater response dispersion, alternative scaling approaches, or complementary qualitative methods to better capture subtle variations in communication behaviors.

## Conclusions

Overall, patients have a positive perception of family physicians' communication behaviors; however, it is especially important to adopt an active attitude of “giving the patient a voice”. It is never too much to remember that communication is a dynamic process that depends on two interlocutors, in which the five domains of physician-patient communication, challenge, encouragement and praise, nonverbal support, understanding and friendly relationship, and control, should be privileged. In the communication process, it is essential that there is a sharing of expectations between the physician and the patient in each consultation and that the patient's right to autonomy is always guaranteed. 

In conclusion, communication is not just an extra part of medical care; it is central to it.

## Appendices

Appendix 1. Questionnaire

Communication is the basis of any doctor-patient relationship and is a particularly valuable tool in general practice. It is intrinsically linked to the creation of a relationship of trust between the patient and their doctor.

Communication in this pair is fundamental to understanding and valuing the patient’s beliefs and fears and including them in medical decision-making. The fact that they feel listened to and have an active role in their health makes the relationship between doctor and patient stronger and predisposes them to have more confidence in the clinician’s attitudes and therapeutic plan.

Several factors have been documented as influencing the quality of communication in the doctor-patient duo. However, there is a lack of evidence regarding these factors in the context of the Portuguese population.

Therefore, this research project aims to assess the perception of users regarding the doctor’s communicative behavior and explore differences according to various demographic and socioeconomic factors in the Portuguese population.

Informed consent

We invite you to take part in a scientific research study conducted by a group of general practitioners from the Regional Health Administration of the North and Center.

The scope of the research project focuses on users’ perceptions of the doctor’s communicative behavior.

As a participant, you will only need to answer this questionnaire, which will take you an average of 6 minutes to complete. There are no right or wrong answers, what matters is what you really think and feel. It is important that you read carefully and answer all the questions.

The questionnaire is anonymous and confidential. You can stop taking it at any time and without any prejudice.

The data obtained will subsequently be subject to joint statistical analysis, after being placed in a coded database, i.e. without there being any knowledge of who answered or how they answered.

By answering the questionnaire, you are authorizing the collection, processing, and analysis of the data provided, in accordance with and for the purposes of this research.

In this sense, the data will be used exclusively for scientific research purposes.

If you have any questions while filling in the questionnaire or need further clarification, please do not hesitate to contact the research team via e-mail at comunic.imgf@gmail.com or your GP or family nurse.

Your participation is voluntary but essential.

Thank you for your contribution!

The Research Team,

Bárbara Moreira, Catarina Alves Gonçalves, Catarina Falcão Alves, Catarina Sousa Alves, Ludovina Rocha and Rita Pessoa Coutinho.

1. Do you agree to take part in our study? 

If you agree, please mark the following sentence.

◯      Yes, I AGREE to take part in the study and to have my data processed, after reading the consent form above.

2. If you would like to receive the results of our study, please provide us with your e-mail address.

______________________________________

Your answer to this question is optional.

Your e-mail address will be separated and placed in an external database, in relation to the other data in this questionnaire, for the analysis process.

Coding

3. To avoid duplicates, please create a six-character code with the following parameters:

- two digits of your birthday;

- the first two letters of the place where you were born;

- the first two letters of your mother’s name.

_____________________________

As an example, you should get a code like this 08PACA.

**Table 4:** Part I: Sociodemographic Data

Question	Answer(s)
4. Age	____ years-old
5. Gender (mark only one option)	Male
	Female
	Other: _____________
6. Marital status (mark only one option)	Single
	Married
	Cohabiting
	Divorced
	Separated
	Widowed
7. Education level (mark only one option)	Illiterate
	Primary School
	Elementary School
	Middle School
	High School
	Higher Educations
8. Professional status (mark only one option)	Employed
	Self-employed
	Employer
	Unemployed
	Student
	Retired
	Other: _________________________
9. Profession/Professional area or field of study (please answer this question even if you are retired or unemployed)	__________________________________
10. Area of residence	__________________________________

**Table 5:** Part II: Consultation Data

Question	Answer(s)
11. Type of consultation (mark only one option)	Scheduled/Programmed appointment
	Acute disease appointment
12. Gender of the doctor who carried out the consultation (mark only one option)	Male
	Female
13. Was this your first consultation with this doctor? (mark only one option)	Yes
	No
14. The consultation was carried out by (mark only one option)	Your family doctor
	Another doctor from your Health Unit
	Resident doctor
15. For how long have you been seen by your GP? (mark only one option)	Less than 1 year
	Between 1-3 years
	Between 3-5 years
	More than 5 years
16. Do you have any illnesses that require you to see your GP every 6 months for follow-up (e.g. high blood pressure, diabetes)? (mark only one option)	No
	Yes

Part III:  Doctor communication behavior questionnaire (DCBQ-QCCM) [3]

The questions in this section of the questionnaire relate to the interactions you have with your GP during consultations. You should answer how often each interaction takes place.

If the consultation was carried out by an intern/trainee, answer on the basis of this doctors’ behavior and not that of your GP.

To answer, think about the consultation you have just had.

Remember that there are no right or wrong answers, what matters is what you really think or feel. Answer calmly.

For each question, choose only one of the answer alternatives:

1 - If the practice happens NEVER

2 - If the practice happens RARELY

3 - If the practice happens SOMETIMES

4 - If the practice happens FREQUENTLY

5 - If the practice happens ALWAYS

**Table 6:** Part III.1: Challenge Domain

Questions	Answer(s)				
	Never	Rarely	Sometimes	Frequently	Always
17. Does your doctor ask you questions to confirm that you have understood your problem?	1	2	3	4	5
18. Does he/she ask you to explain the proposed treatment in your own words?	1	2	3	4	5
19. Does he/she encourage you to follow the proposed treatment?	1	2	3	4	5

**Table 7:** Part III.2: Encouragement and Commendation Domain

Questions	Answer(s)				
	Never	Rarely	Sometimes	Frequently	Always
20. Does your doctor ask for your opinions during consultations?	1	2	3	4	5
21. Does he/she praise you when you comply with treatment recommendations?	1	2	3	4	5
22. Does he/she adapt the treatment to your routine/reality?	1	2	3	4	5

**Table 8:** Part III.3: Understanding and Friendly Relationship Domain

Questions	Answer(s)				
	Never	Rarely	Sometimes	Frequently	Always
28. Does your doctor trust you?	1	2	3	4	5
29. Does he explain again what you didn't understand?	1	2	3	4	5
30. Does he/she listen to you if you have something to say?	1	2	3	4	5
31. Does he/she notice when you don't understand something?	1	2	3	4	5
32. Is he/she patient with you?	1	2	3	4	5
33. Is he/she friendly with you?	1	2	3	4	5
34. Does he/she care about you?	1	2	3	4	5
35. Is he/she someone you can count on?	1	2	3	4	5
36. Does he/she use a language you understand?	1	2	3	4	5
37. Does he/she care about your fears and anxieties?	1	2	3	4	5

**Table 9:** Part III.4: Control Domain

Questions	Answer(s)				
	Never	Rarely	Sometimes	Frequently	Always
38. Does your doctor let you give your opinion on the treatment plan?	1	2	3	4	5
39. Is he/she willing to change the treatment plan if you are unable to carry it out?	1	2	3	4	5

The questionnaire is over!

Thank you for your attention and for answering our questions.

Your contribution was essential to our work.

We would like to remind you that we are available for any further questions regarding this study by e-mail: comunic.imgf@gmail.com.
